# Alopecia universalis with IL-12-RB1 and STAT4 mutations effectively treated with upadacitinib

**DOI:** 10.1016/j.jdcr.2024.08.005

**Published:** 2024-08-30

**Authors:** Emily R. Gordon, Luke Horton, Natasha A. Mesinkovska

**Affiliations:** a Columbia University Vagelos College of Physicians and Surgeons, New York, New York; b Department of Dermatology, University of California, Irvine, Irvine, California

**Keywords:** alopecia areata, alopecia universalis, genetic sequencing, IL-12, JAK inhibitors, STAT4, upadacitinib

## Introduction

Although alopecia areata (AA) is among the most common pediatric dermatologic disorders, there are few effective Food and Drug Administration-approved treatments for patients <12 years; especially for severe AA such as alopecia totalis or alopecia universalis (AU). As the JAK-STAT pathway is implicated in AA pathogenesis, Janus kinase inhibitors (JAKi) are increasingly prescribed. Ritlecitinib was approved for patients >12 years and baricitinib was approved for patients ≥18 years with severe AA. However, effectiveness in younger children is understudied, and it is unclear which patients will respond to JAKi. We present a 9-year-old boy with AU and atopic dermatitis (AD), with mutations in IL-12-RB1 and STAT4, who had full hair regrowth after 6 months of upadacitinib. This report highlights the role of genetic testing in children with severe, early-onset disease and suspected immunodeficiencies. It demonstrates upadacitinib as a potentially effective treatment for children with recalcitrant AU.

## Case report

A 9-year-old boy with AD, seasonal allergies, asthma, and reported history of erythema-multiforme-like vaccine reactions presented to clinic for hair loss. The patient’s mother had Hashimoto thyroiditis, and tested positive for antinuclear and anticardiolipin antibodies. One year before presentation, the patient experienced a patch of alopecia after an influenza infection. He was prescribed clobetasol solution by an outside dermatologist and experienced hair regrowth. Several months later, after a family trip, the patient experienced rapid-onset complete hair loss. He was treated with 1-month of prednisone, squaric acid, topical steroids, and phototherapy, without improvement.

**Fig 1:**
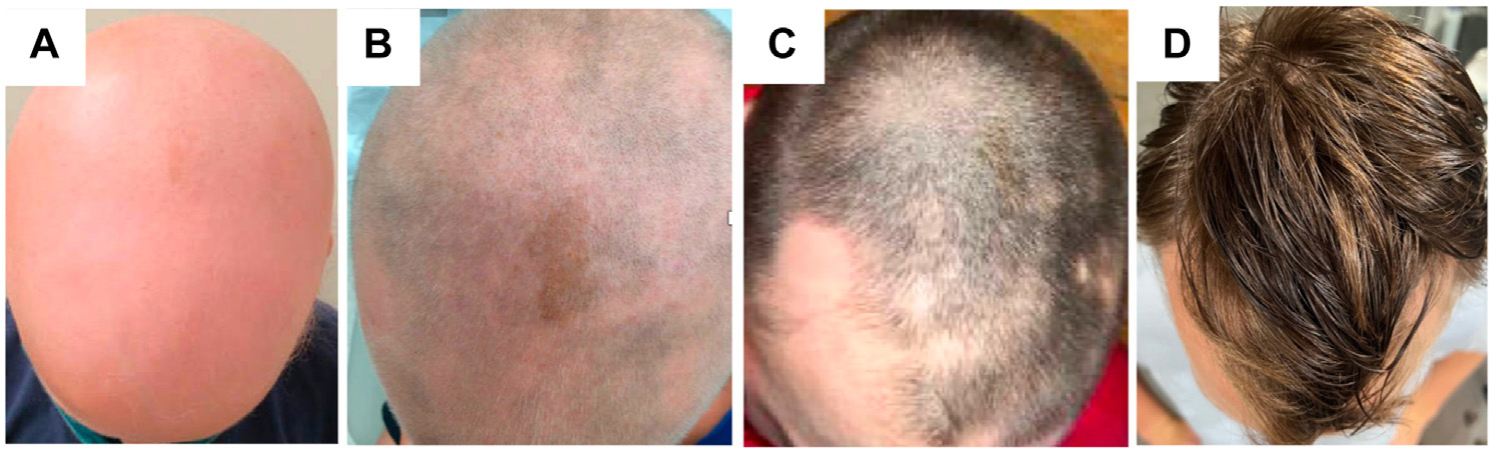
Clinical Initial presentation (**A**), after several months on dupilumab (**B**), after 3 months on upadacitinib (**C**), and after 1 year on upadacitinib (**D**).

Upon presentation to our clinic, his examination demonstrated AU, Severity of Alopecia Tool score 99%, without erythema, folliculitis, scale, or hair regrowth (Fig 1, *A*). He had thinning of the bilateral eyebrows and lashes with nail pitting. He had scaly erythematous plaques on the back of the legs. His laboratory results showed elevated double-stranded DNA, IgE, eosinophils, tryptase, and CTLA. Evaluation for primary immunodeficiencies (Invitae panel, Invitae) was performed because of his age, disease severity, and personal and family history suggesting immune system dysregulation. It revealed several mutations, notably IL-12-RB1 c.733G>T (p.Val245Leu) and STAT4 c.650C>T (p.Thr217Ile). The patient was prescribed dupilumab and started oral antihistamines, with improvement in his AD and asthma. There was growth of fine hairs on the crown of the scalp and regrowth of the right eyelashes (Fig 1, *B*). After 1 year of treatment, the patient’s insurance denied coverage for further dupilumab. The patient initiated 2.5 mg of minoxidil, without improvement in hair growth in 3 months. Then the patient started upadacitinib 15 mg daily. After 3 months, the patient had significant hair regrowth on the scalp, eyebrows, and eyelashes (Fig 1, *C*). Because of parental concerns, the patient was tapered to 15 mg 4 times a week. After 6 months of therapy, the patient experienced near-full hair regrowth, including scalp (Severity of Alopecia Tool score 98%), eyelashes, and eyebrows, without laboratory abnormalities or adverse effects, and has maintained regrowth for over 1 year (Fig 1, *D*).

## Discussion

AA is the third most common dermatologic issue in children.^1^ It has a significant effect on quality of life, especially in severe cases, which are often treatment-refractory.^2^ Therapeutic options are limited in younger patients because of their lack of inclusion in clinical trials and heightened concern for adverse effects. Mainstays of treatment include corticosteroids, immunosuppressants, vasodilators, or sensitizers, which have limited efficacy. Recent approval of the JAKi ritlecitinib and baricitinib is promising for severe AA; however, they are understudied in younger children.

Most data concerning JAKi in children are from tofacitinib, a JAK1 and JAK3 inhibitor.^3^ Children 4 to 10-years-old had some hair regrowth on tofacitinib with minimal side effects.^4^^,^^5^ Upadacitinib is a JAKi preferentially inhibiting JAK1. Because of its favorable safety profile and clinical response, it has been approved for patients with AD ≥12-years-old.^6^ There are emerging reports supporting use of upadacitinib for AA and clinical trials are underway.^7^^,^^8^ Of the published cases, almost all had concurrent AD, demonstrating the potential role of atopy and inflammation in AA pathogenesis. There are only 2 published pediatric cases of AU demonstrating the effects of upadacitinib.^7^^,^^8^ In one, a 9-year-old girl with a 7-year history of AU and AD, refractory to topical steroids, minoxidil, tacrolimus, and oral glycyrrhizin, was started on 15 mg of upadacitinib daily with hair regrowth in 6 weeks and maintained response at her 5-month follow-up visit, without adverse effects.^7^ Kołcz et al^8^ described a 14-year-old girl with AU who failed topical minoxidil, mometasone furoate, diphenylcyclopropenone, and phototherapy. On 15 mg of upadacitinib daily, she had resolution of AD and full hair regrowth on the scalp, eyebrows, and eyelashes. She experienced mild leukopenia which self-resolved.^8^

Mutational profiles of children with AU are largely unexamined but may demonstrate therapeutically targetable mutations such as in this case with a mutation in the JAK-STAT pathway. Evaluation for primary immunodeficiencies in our patient revealed mutations in IL-12-RB1 and STAT4. Interleukin 12 (IL-12) is a cytokine that contributes to differentiation of naive T-cells into T helper 1 (Th1) cells, which are crucial for cell-mediated immunity.^9^ The patient’s mutation in the IL-12-RB1 subunit may affect downstream IL-12 signaling pathways and augment CD8 T-cell responses and differentiation of naive CD4 T-cells into Th1 effectors. STAT4 is a transcription factor that mediates responses to IL-12 and IL-23, promoting the differentiation of Th1 and Th17 cells, respectively, which contribute to production of proinflammatory cytokines.^10^ Our patient’s mutation in IL-12R and STAT4 may have led to increased Th1 and Th17 responses and IL-12 and IL-23 receptors, activating JAK2 and tyrosine kinase 2 and leading to phosphorylation of STAT4. By inhibiting JAK1, and to a lesser extent JAK2 and tyrosine kinase 2, upadacitinib may modulate the IL-12 and STAT4 pathways, and reduce these autoimmune and inflammatory responses.^6^ Thus, we hypothesize that this patient’s mutations may reflect an increased responsiveness to JAKi, however, additional investigation into this pathway is needed.

We present a rare case of AU in a young man with IL-12-RB1 and STAT4 mutations, who had full hair regrowth on upadacitinib. Previous reports of patients with AU treated with upadacitinib have not included mutational testing. With increased accessibility and reduced cost of genetic tests, dermatologists should consider them for young patients with severe AA who may benefit from targeted therapies. Further investigation into genetic profiles of children with AU may demonstrate additional therapeutic targets and elucidate origins of immune processes regulating this condition.

## Conflicts of interest

None disclosed.
